# 3D-Printed Microfluidic One-Way Valves and Pumps

**DOI:** 10.3390/mi14071286

**Published:** 2023-06-23

**Authors:** Hunter Hinnen, Matthew Viglione, Troy R. Munro, Adam T. Woolley, Gregory P. Nordin

**Affiliations:** 1 Department of Electrical & Computer Engineering, Brigham Young University, Provo, UT 84602, USA; 2 Department of Mechanical Engineering, Brigham Young University, Provo, UT 84602, USA; 3 Department of Chemistry & Biochemistry, Brigham Young University, Provo, UT 84602, USA

**Keywords:** microfluidics, 3D printing, one-way valve, microfluidic pump, microfluidic mixer

## Abstract

New microfluidic lab-on-a-chip capabilities are enabled by broadening the toolkit of devices that can be created using microfabrication processes. For example, complex geometries made possible by 3D printing can be used to approach microfluidic design and application in new or enhanced ways. In this paper, we demonstrate three distinct designs for microfluidic one-way (check) valves that can be fabricated using digital light processing stereolithography (DLP-SLA) with a poly(ethylene glycol) diacrylate (PEGDA) resin, each with an internal volume of 5–10 nL. By mapping flow rate to pressure in both the forward and reverse directions, we compare the different designs and their operating characteristics. We also demonstrate pumps for each one-way valve design comprised of two one-way valves with a membrane valve displacement chamber between them. An advantage of such pumps is that they require a single pneumatic input instead of three as for conventional 3D-printed pumps. We also characterize the achievable flow rate as a function of the pneumatic control signal period. We show that such pumps can be used to create a single-stage diffusion mixer with significantly reduced pneumatic drive complexity.

## 1. Introduction

Microfluidic devices have many applications in the biomedical, biological, chemical, and life science fields [1,2,3]. Recent advances in 3D printing for microfluidics have made it possible to create devices of increasing complexity and decreasing size [4,5,6,7,8,9,10,11,12]. Unlike traditional lithographic methods that can be time-consuming and require the use of a cleanroom, 3D printing requires little manual labor and minimal resources beyond a 3D printer. Microfluidic devices that are 3D-printed also have fewer geometric constraints than those made with fabrication methods using stacked and bonded 2D layers. Designs not constrained by such planar geometries can be more compact and have unique features and functionality. Developing new devices for 3D printing helps to narrow the gap between scalable digital light processing stereolithography (DLP-SLA) for microfluidics and well-developed but less convenient methods like soft lithography.

Nonlinear microfluidic structures are useful for achieving precise fluidic control within a device [13]. Standard microfluidic channels with a constant cross section have fixed fluidic resistance such that the pressure drop across a channel increases linearly with increased flow rate. By deliberately introducing compliant structures to a microfluidic device, such as flexible membranes, the pressure–flow rate relationship can be made nonlinear and, therefore, asymmetric with respect to flow direction [14]. By leveraging this phenomenon, we can create one-way valves that can seal passively based on the direction and magnitude of flow.

Using DLP-SLA, we developed three different microfluidic one-way valves, also known as check valves. To create these components, we required a 3D printer capable of fabricating (1) channels small enough to produce significant fluidic resistance and (2) compliant mechanisms such as membranes. A nonlinear fluidic structure results when the pressure difference across a small channel for a given flow rate is greater than or equal to the pressure required to deflect a membrane within the structure. With larger channels or stiffer membranes, it is still possible to fabricate a working check valve; however, the flow rate within the device must be much greater, which may be unsuitable for some applications.

Multiplexed microfluidic devices with many active components, such as for lab-on-a-chip applications, can benefit greatly from reduced pneumatic drive complexity. To reduce the number of pneumatic inputs, some active components within a device can be replaced with nonlinear passive components. For example, passive one-way valves can be used to govern flow direction without actively actuating control valves to close off channels [15]. They can also be used with a displacement chamber to create micropumps that require a single cyclic pneumatic input [16]. In addition to user simplicity, the reduced number of pneumatic control channels within a device allows for even more compact designs. The one-way valves we fabricated enable complex 3D-printed lab-on-a-chip devices that are both easy to use and have a smaller footprint.

## 2. Materials and Methods

### 2.1. Custom 3D Printer

The 3D printer we used to fabricate devices is based on a Visitech LRS-WQ light engine with a projected image size of 2560 × 1600 pixels. Each pixel has a pitch of 7.6 μm, making the total build area 19.5 mm × 12.2 mm. We used a 365 nm LED with a short pass filter to polymerize our custom resins. The build platform is controlled by a 100 mm linear Griffin Motion stage with a single-axis Galil controller. The resin tray has a glass bottom covered with a nonstick FEP (fluorinated ethylene propylene polymer) film. The 3D printer is controlled by custom Python software designed to give the user full control over parameters such as exposure time; build platform positioning, velocity, and acceleration/deceleration; and various wait times on both a layer-by-layer and pixel-by-pixel basis.

We can create compliant mechanisms within a monolithic print by controlling the UV dose and layer thickness for specific pixels during a print run. Exposing multiple images at lower doses and for locally arbitrary layer thicknesses makes this precise control over the 3D optical dose distribution possible [4]. For example, by modifying software parameters and the projected image stack, we can reduce the exposure time to a circular region of pixels to make a flexible membrane between stacked cylindrical chambers. The ability to maintain chip strength in the bulk without overexposing void features also allows us to reduce feature sizes [8]. The smallest feature in our one-way valves, which is a narrow channel that is 20 μm tall (i.e., two 10 μm layers), is made possible by these methods. Unless otherwise noted, all devices were printed with 10 μm thick layers. Ref. [17] includes an in-depth analysis of achievable feature fidelity in 3D-printed structures for microfluidics.

### 2.2. Materials

We used a custom resin that was formulated to minimize the cross-sectional area of channels and other microvoids such as valve fluid chambers [8]. When fabricating a device layer by layer, optical penetration from subsequent layers can polymerize the resin within channels and other voids of previous layers. To avoid this, we added a photoabsorber to our resin that limited the penetration depth of the spectrally filtered 365 nm LED to ~1.3 times our layer height of 10 µm [7]. The resin we used to fabricate the devices (with valves) consisted of a poly(ethylene glycol) diacrylate (PEGDA, MW258) monomer containing 2% (*w*/*w*) 2-nitrophenyl phenyl sulfide (NPS) photoabsorber and 1% (*w*/*w*) phenylbis(2,4,6-trimethylbenzoyl)phosphine oxide (Irgacure 819) photoinitiator. All devices reported in this paper were printed with this resin formulation and consisted solely of this material, including the membranes in each type of one-way valve.

### 2.3. Post-Print Processing

After printing each device, unpolymerized resin occupied the intended void regions, which had to be removed prior to the post-exposure optical cure step. Our method for clearing the printed devices involved flushing the unpolymerized resin out of the device by applying a vacuum, followed by the injection of isopropyl alcohol (IPA) into the channels and flushing a second time. This process normally requires each intended fluidic void to connect to at least two input/output ports to facilitate the flow of unpolymerized resin toward the applied vacuum. However, we found that if we let a just-printed chip sit for 24 to 48 h before flushing, in many cases, unpolymerized resin in dead-end channels absorbs into the polymerized bulk material, resulting in a self-clearing process. This self-clearing process allows for greater design flexibility by permitting dead-end channels in selected regions. After self-clearing, we flushed the channels with IPA and then optically cured with a 430 nm LED at a measured irradiance of 11.3 mW/cm^2^ in the curing plane for 20 min to drive further polymerization to increase membrane toughness.

## 3. Results and Discussion

### 3.1. One-Way Valve Design

Each of the three one-way valve designs has a membrane that can be deflected up or down based on flow direction. When referring to the fluid flow direction, a “forward” flow deflects the membrane out of the way of the fluid path, resulting in a quasi-linear pressure–flow rate relationship for a one-way valve similar to that in a standard microfluidic channel. “Reverse” fluid flow deflects the membrane to obstruct the fluid path, breaking the linearity between flow rate and pressure. For an ideal one-way valve, any reverse flow causes the channel to seal off completely such that the flow rate is zero. However, actual membranes require a minimum force to actuate before sealing. Moreover, some nonlinear fluidic element geometries do not create a perfect seal, which results in residual fluid leakage. Each of our one-way valves exhibits unique forward/reverse flow characteristics that can be used for different applications. In each case, they function as a fluidic diode, with a greater or lesser degree of reverse bias fluid leakage.

In addition to small fluid leakage and low actuation force, a one-way valve will ideally have a small internal volume and be able to withstand relatively high fluid pressure (we successfully tested up to 50 psi for all three designs). Because polymerized PEGDA has a relatively high Young’s modulus of about 130 MPa, it is more difficult to create a valve that seals completely against the bottom of a channel. However, it also means that the membrane has a higher restoring force and does not require a reverse pressure to open after sealing [18]. By modifying the three-dimensional geometry of each design, we can create better seals and reduce the valve internal volume.

**Figure 1:**
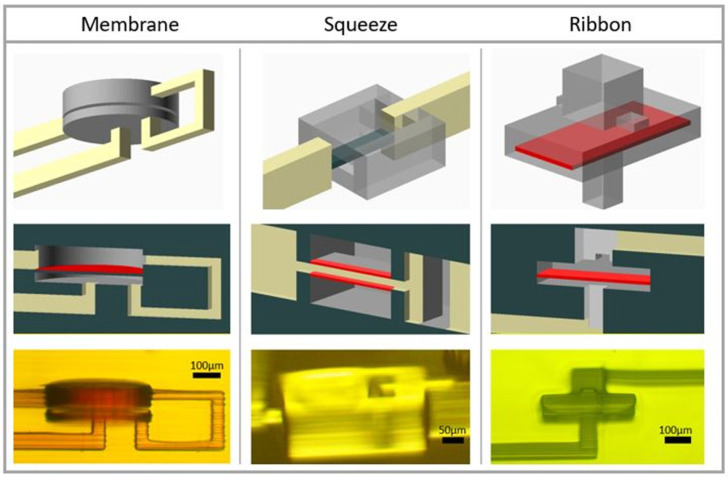
Membrane (**left**), squeeze (**center**), and ribbon (**right**). One-way valve CAD designs (**top**) with membranes emphasized in red (**middle**) and microscope images of corresponding 3D-printed components (**bottom**).

The three one-way valve designs are illustrated in Figure 1 and discussed below. Each design was fabricated with a layer exposure time of 325 ms for the bulk regions and a reduced exposure of 260 ms for membranes (indicated in red in Figure 1). Example one-way valve test chips are shown in Supplemental Information Figure S1.

#### 3.1.1. Membrane Valve

**Figure 2:**
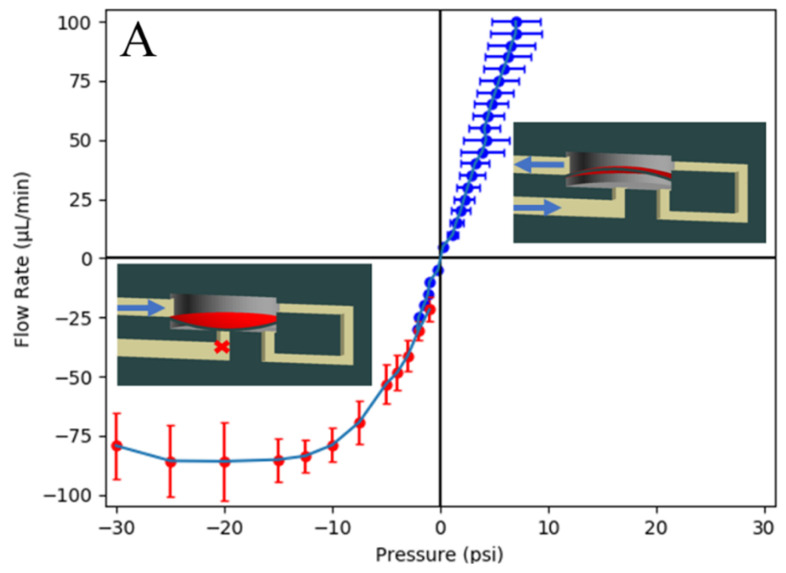

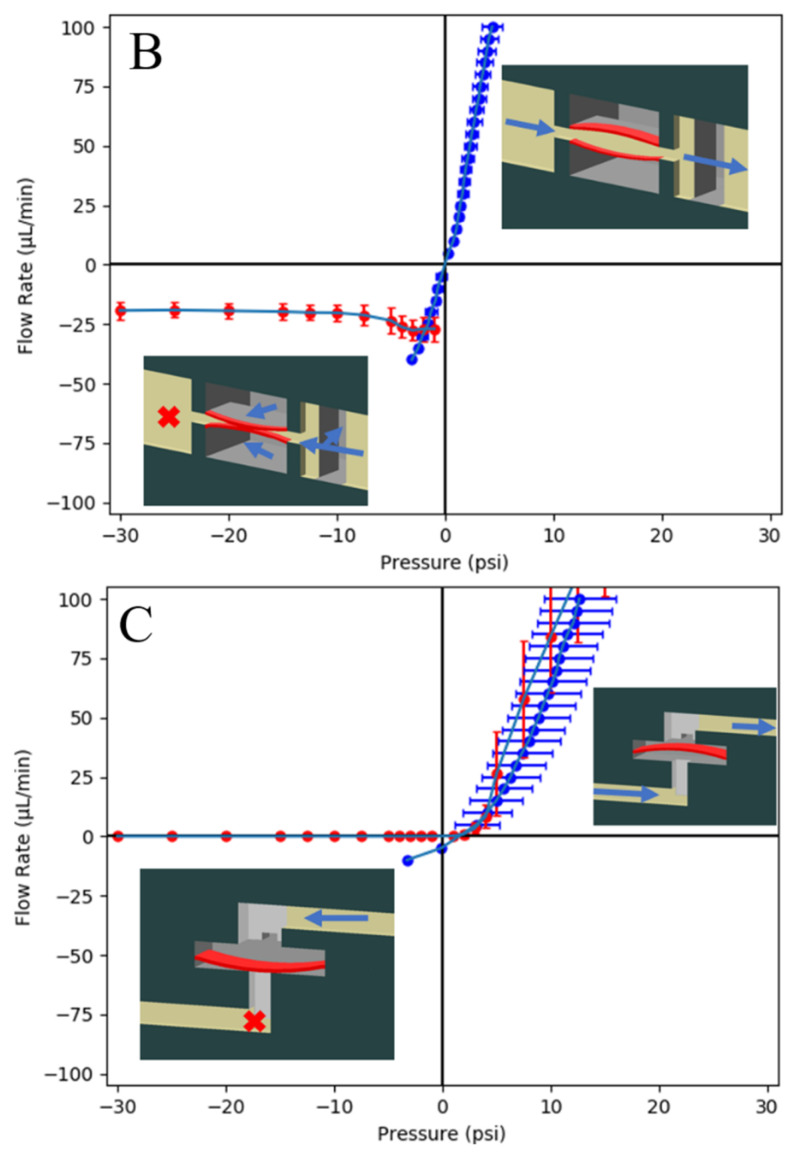
Measured average pressure and flow rate relationships for (**A**) membrane, (**B**) squeeze, and (**C**) ribbon one-way valves with corresponding CAD illustrations of membrane deflection based on flow direction. Blue arrows indicate fluid flow direction with corresponding membrane deflection denoted in red. Red “X” denotes reduced flow for reverse flow case. Positive flow rate is forward flow, and negative flow rate is reverse flow. Data in blue depict measuring pressure as a function of flow rate. Data in red entail measuring flow rate as a function of pressure. Data points are an average of 7 valves for each type, and error bars denote ±1 standard deviation.

The membrane one-way valve (Figure 1 left) is based on a standard 3D-printed membrane valve [6]. A circular membrane acts as a divider between two cylindrical chambers. The membrane is 380 µm in diameter and was printed as two 5 µm tall layers. The high resistance channel that connects the upper and lower chamber is 46 µm × 30 µm and creates a pressure drop that increases with flow rate. Depending on flow direction, the difference in chamber pressures will either deflect the membrane out of the way of the fluid path (Figure 2A inset right), which is the forward fluid direction, or will close off the channel that the fluid would exit through (Figure 2A inset left), which is the reverse fluid direction. The lower chamber is 40 µm tall, which is the distance that the membrane must deflect to seal off the outlet channel. The volume of the entire valve is 10.3 nL.

#### 3.1.2. Squeeze Valve

The squeeze one-way valve (Figure 1 center) operates similarly to a standard membrane valve but has a membrane both above and below a thin 20 µm tall channel, pinching the channel closed for fluid flow in the reverse direction [4]. Fluid flow through this high-resistance channel experiences a pressure drop according to the Venturi effect. For fluid flow in the reverse direction (Figure 2B inset left) the pressure preceding this channel propagates around membranes that make up the top and bottom of the channel, pinching them closed. Each membrane is 61 µm × 182 µm and is 5 µm thick, and each must deflect 10 µm to seal off the channel. Propagation in the forward direction (Figure 2B inset right) causes the membranes to deflect so as to expand the thin channel. The volume of the entire valve is 10.2 nL.

#### 3.1.3. Ribbon Valve

Unlike the previous two designs, the ribbon one-way valve (Figure 1 right) uses flow-based membrane deflection to create nonlinearity. In the forward direction, the ribbon membrane deflects upward where the channel leading out of the valve is widened to create an exit for fluid that goes around both sides of the ribbon membrane (Figure 2C inset right). In the reverse direction, the channel is narrower than the ribbon, such that the deflected ribbon membrane blocks flow entirely (Figure 2C inset left). The ribbon membrane is 167 µm × 365 µm, 10 µm thick, and fixed at its two narrow ends with 30 µm of clearance on each side of its long dimension. The volume of the entire valve is 5.42 nL.

### 3.2. One-Way Valve Characterization

To characterize each valve type, we measured flow rate as a function of pressure and vice versa. In the forward direction (upper right quadrant in Figure 2), we measured the pressure drop across the one-way valve once it reached a steady state for a set of constant flow rates [19]. As expected, each one-way valve design had a quasi-linear flow rate/pressure characteristic such that the valve behaved similar to a standard microfluidic channel. In the reverse direction (lower left quadrant in Figure 2), we pressurized a fluid reservoir, connected it to the one-way valve, and measured the fluid movement through a piece of tubing with a known inner diameter as a function of time to calculate the flow rate. (Note: if we had used a constant flow rate instead, the resulting pressure would build until device failure.) Using these two methods allowed for high-resolution steady-state measurements in both forward and reverse flow directions.

Typical one-way valve measured flow rate/pressure curves, similar to electrical diode I-V curves, are shown in Figure 2. For the membrane one-way valve in Figure 2A, note the nearly linear relationship between approximately −8 psi and >10 psi, indicating that over this pressure range, membrane deflection does not significantly impede fluid flow. However, once the pressure is near −10 psi, membrane deflection limits the reverse flow rate. Out of the three designs, this one had the largest reverse leakage flow. To seal completely, this valve would require a pressure significantly above our standard microfluidic operating pressures, which are typically <30 psi. The reverse leakage flow could be reduced by decreasing the height of the lower chamber, but as explored later, integrating this valve into a pump does not require the valve to seal perfectly. An example video of membrane one-way valve operation is shown in Supplemental Video S1 and described in the Supplemental Information.

The squeeze one-way valve measured flow rate/pressure relationship is shown in Figure 2B. Note that it has a more attractive flow/pressure relationship compared to the membrane one-way valve in that a reverse flow pressure of approximately −3 psi results in enough membrane deflection to restrict the reverse flow rate. For pressures less than −3 psi, the squeeze one-way valve acts as a flow regulator with a flow rate of ~25 µL/min [20]. This leakage flow is substantially smaller than for the membrane one-way valve.

The measured ribbon valve flow rate/pressure curve is shown in Figure 2C and is nearly ideal in its characteristics. There is no flow in the reverse direction for any negative pressure. Moreover, the data also suggest that this valve is normally closed since it requires ~2 psi in the forward direction for fluid to begin flowing. This is likely due to the ribbon membrane sticking slightly to the bottom of the main chamber during post-print clearing.

### 3.3. Single Pneumatic Control Pumps

**Figure 3:**
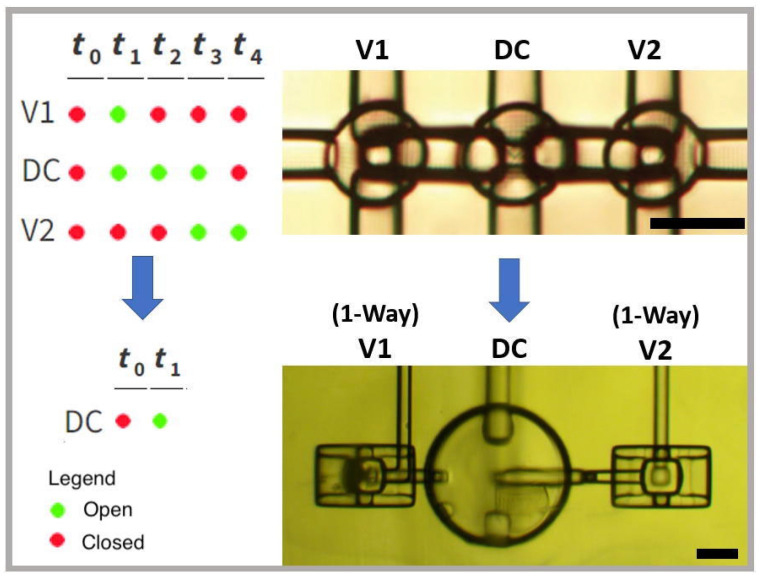
Pump timing logic and top-view microscope images for (**top**) conventional membrane valve pump and (**bottom**) single pneumatic control pump based on one-way valves. Red: actuated (pressure applied; valve closed). Green: not actuated (atmospheric pressure or vacuum applied; valve open). Black scale bar in images is 150 µm. Upper right image adapted from Ref. [21] with permission from The Royal Society of Chemistry.

Typical microfluidic pumps require at least three independently operated pneumatic valves, arranged as valves V1 and V2 in Figure 3 sandwiching a displacement chamber (DC); the DC can be an actual valve that can fluidically close, or a valve-like structure that only displaces fluid [21]. A five-phase actuation cycle is used to draw fluid through V1 and expel it through V2 to create a pulsatile pumping action [21]. The actuation cycle begins at time t_0_, with each valve and DC in a closed state containing a minimum volume of fluid. At t_1_, V1 and DC open, drawing fluid into the DC fluid chamber through V1. At t_2_, V1 closes to isolate the fluid in the DC. At t_3_, V2 opens, followed by the DC closing and expelling fluid at t_4_. The cycle then repeats with V2 closing such that the system returns to the state at t_0_.

Our one-way valves reduce the drive complexity of pumps by requiring a single pneumatic input instead of three. This facilitates a two-phase operating cycle as shown in Figure 3. At t_0_, the DC (in this case implemented as an actual valve) is actuated. Because of the nonlinear flow behavior of the one-way valves, more expelled fluid exits the pump through V2 than V1, setting up a net flow through V2. When the DC pneumatic pressure is released or vacuum is applied, more fluid is pulled through V1 than V2 to fill the DC fluid chamber. Because both one-way valves are oriented in the same direction, the repeated actuation of the DC creates a pulsatile flow in the forward direction [22]. This design reduces the total cycle time by 60% for a given phase interval, Δt = t_i_ − t_i−1_. Example pump test chips are shown in Supplemental Information Figure S1 and the fluid flow generated by a membrane valve pump is shown in Supplemental Video S2.

### 3.4. Pump Designs

**Figure 4:**
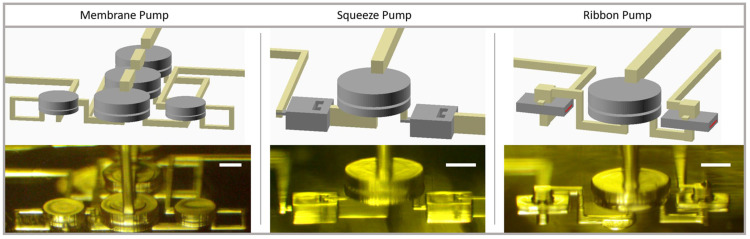
CAD model (**top**) and microscope photograph (**bottom**) of single pneumatic input pump designs using each type of one-way valve. Scale bars are 200 µm.

Each one-way valve design has been successfully used with a displacement chamber to create an effective pump. CAD designs and corresponding fabricated device photographs are shown in Figure 4. In all cases, the DC is a standard pneumatic membrane valve that is 532 µm in diameter and has a 10 µm thick membrane and 60 µm tall fluid chamber. We chose to use a valve rather than a valve-like structure that only displaces fluid so that when it is actuated, the pump as a whole can act as a closed valve to prevent fluid flow, which is a convenient additional capability for a microfluidic pump. Note that the membrane one-way valve pump design (Figure 4 left) uses three DCs in parallel to generate the required displaced fluid volume get the one-way valves into their nonlinear operating range. The other two pump designs operate well with a single DC. Even though the membrane and squeeze one-way valves do not seal completely during pumping, the resistance in the reverse direction is higher than in the forward direction, creating a net positive flow. The use of three DCs for the membrane valve pump limits its use to higher-flow-rate applications. For lower-flow-rate applications, one of the other two designs may be more appropriate, although they also can be adapted to high-flow-rate applications by using more DCs in parallel to displace a greater volume in each pump cycle. Note that the use of multiple DCs does not increase the number of required pneumatic control channels as all DCs can be run in parallel from the same pneumatic line.

### 3.5. Pump Characterization

**Figure 5:**
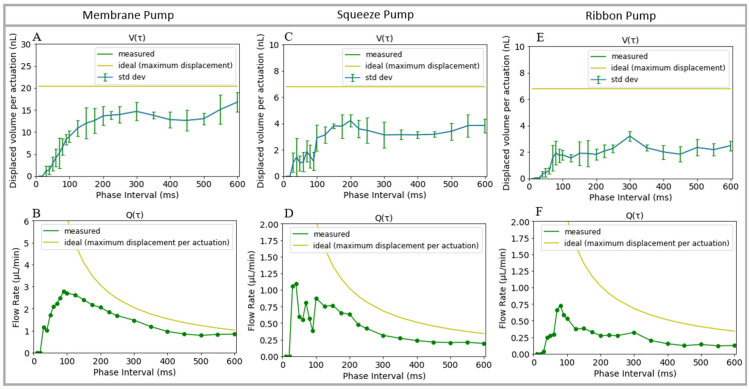
Displaced volume per actuation of the displacement chamber (**A**,**C**,**E**) and flow rate (**B**,**D**,**F**) for each pump design as a function of phase interval. (**A**,**C**,**E**) Average of 7 to 13 pump cycles required to fill a serpentine channel of known volume for each pump type, with error bars denoting ±1 standard deviation in the net volume per pump cycle.

To characterize each pump design, we used video analysis to calculate the volume of dyed water pumped through a channel of known cross-sectional area. By sampling at the phase interval, Δt, we can determine the volume of water that is displaced per actuation of the DC for each pump design, as shown in Figure 5A,C,E. An example video for a membrane pump is included in the Supplemental Information. We can also average the change in volume over time to determine the average volumetric flow rate generated by each pump (Figure 5B,D,F). For an ideal pump, the displaced volume per actuation can be approximated as the volume of the DC’s fluid chamber (~7 nL) multiplied by the number of DCs used in the design. In practice, the actual displaced volume is less because of leaky one-way valves and because the membrane does not collapse fully into the fluid chamber when it is deflected. Nonetheless, we can estimate the efficiency of a pump as function of the phase interval, Δt, by dividing the measured displaced volume by the approximated ideal displaced volume. Using this estimate, we can see that despite the leakage experienced by the membrane valve at steady state, the membrane pump is more efficient for phase intervals of >100 ms than the other two designs, most likely due to how little resistance the fluid experiences in the forward direction during t_1_.

As seen in Figure 5 bottom row, for each pump, the efficiency gets closer to the ideal maximum flow rate as the pump is driven slower for phase intervals >100 ms. A larger phase interval gives the displacement chamber more time to fill as the membrane relaxes to an undeflected state, but the larger phase interval also reduces the generated flow rate. On the other hand, decreasing the phase interval generally increases the flow rate until the phase interval is so short that there is not enough time to significantly refill the DC. Applying vacuum to help refill the DC generally takes longer than applying pressure to expel fluid, so to further increase flow rate, one could possibly use uneven phase intervals, spending less time in the t_0_ phase and more time in the t_1_ phase.

### 3.6. Single-Stage Diffusion Mixer

Another useful simplification of microfluidic processes is using passive methods to generate mixing between fluids. For example, diffusive mixing can occur by the simple laminar flow of two fluids side-by-side, eliminating the need for active components to agitate the fluids [23]. To dramatically shorten the time needed for diffusive mixing, a high-aspect-ratio 3D-printed channel can be used in which two thin fluid sheets flow in parallel to reduce the distance that a molecular species needs to diffuse, therefore reducing the time it takes to mix the fluids completely [4,24]. As we showed in Ref. [4], combining any number of single-stage 1:1 mixers in series can also create a dose–response assay that has simultaneous dilutions covering multiple orders of magnitude. Passive mixing with high aspect ratio channels in combination with one-way valve-based pumps could make something as complex as a complete on-chip dose–response assay possible with a single pneumatic control input.

### 3.7. Mixer Design

**Figure 6:**
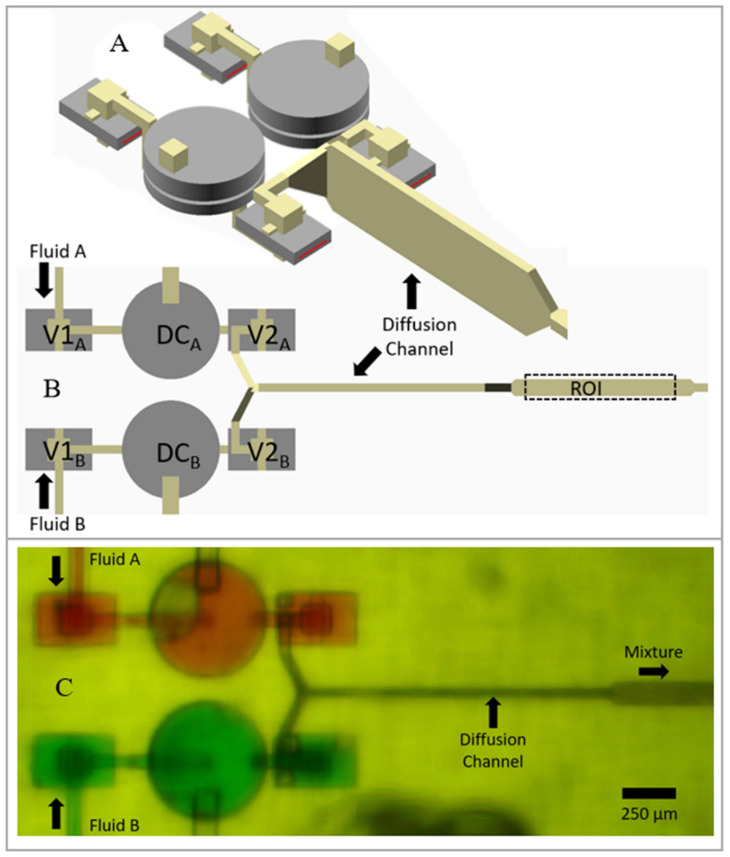
Single-stage 1:1 diffusion mixer using ribbon one-way valves. (**A**) Perspective view, (**B**) top view, and (**C**) micrograph of fabricated device with green and red dye to illustrate the two input fluid paths.

To create this design, we used two one-way valve-based pumps running in parallel and flow both pump outputs into a Y junction that gradually expands vertically and narrows horizontally to match the high-aspect-ratio cross section of the diffusion channel (Figure 6A). We used this junction design rather than a simple T junction because we found that the pulsatile flow from both pumps is likely to cause one fluid to dominate the entrance to the diffusion channel if the flows are opposite to one another. The diffusion channel is 46 µm wide and 370 µm tall, meaning that the molecular species only needs to diffuse 23 µm for mixing to be complete. The principle behind and design of such high-aspect-ratio diffusion mixing channels is discussed in detail in Ref. [4]. We chose to use the ribbon valve version of the pump for this mixer design because it has the least leakage and can run the pumps independently during pump characterization without flowing any fluid backward through the inactive pump, which makes testing more convenient.

When designing the length of the diffusion channel, the total volume of the diffusion channel must be greater than or equal to the volume of the displaced fluid per actuation. If the volume of fluid per actuation exceeds that of the channel, then a portion of the fluid will be pulsed through the diffusion channel without dwelling in it. Additionally, the dwell time in the channel must exceed the diffusion time for a particular diffusion distance and molecular species, as detailed in Ref. [4]. Our diffusion channel has a volume of 20 nL, which is 4 times greater than our displaced volume of ~5 nL (two DCs times the displacement volume given in Figure 5E). This means that our pump cycle time must be no greater than 1/4th of the diffusion time for a chosen molecular species.

### 3.8. Mixer Characterization

**Figure 7:**
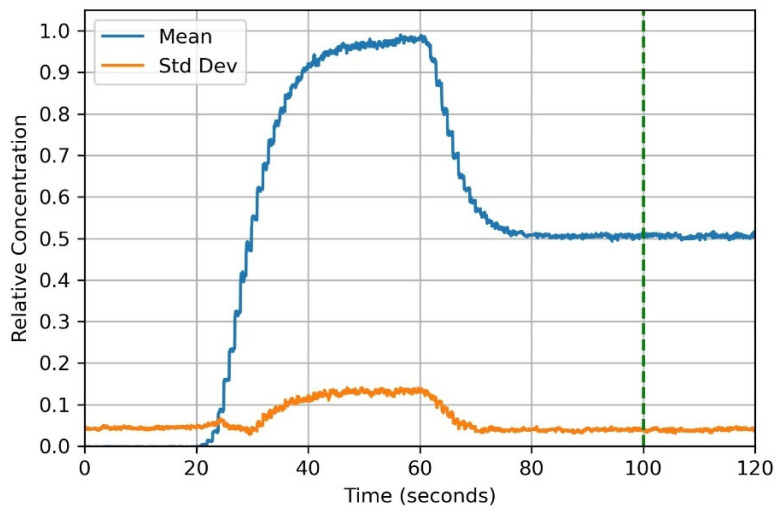
Measured relative concentration for a ribbon valve diffusion mixer. See text for details.

Although both pumps in the mixer can be run with a single pneumatic input, to characterize the pump, we drove them separately to facilitate data collection needed for relative concentration determination. For example, Figure 7 shows the relative concentration of a water-soluble black dye across the analysis region-of-interest (ROI) in Figure 6A after exiting the diffusion channel of the mixer. First, clear water (Fluid A) was pumped to determine the maximum optical transmission through the device channel (0–20 s in Figure 7). Next, water containing black food coloring (Fluid B) was pumped to determine the minimum optical transmission (20–60 s). Lastly, we pumped both fluids simultaneously for 40 s (60–100 s) and then stopped pumping for another 20 s (100–120 s). If the dye has not fully mixed during pumping, the standard deviation of pixel values measured across the ROI will decrease over time. Since the standard deviation remained constant after pumping stops, the 1:1 dilution was well-mixed, as discussed in Ref. [22] (this reference describes the entire measurement process in detail). In our measurements, we also found that there was not an exact linear relationship between the concentration of dye and optical transmission, so we created a calibration curve by measuring optical transmission for known concentrations of food coloring. We used this calibration curve, which is included as Figure S1 in the Supplementary Information, to calculate the relative concentration (Figure 7 vertical axis) for our measured data.

## 4. Conclusions

In this study, we designed and characterized three unique designs for 3D-printed microfluidic one-way valves. Although only the ribbon valve design is reasonably similar to an ideal fluidic diode, the other two designs had advantages, with the membrane valve being used to create more efficient pumps and the squeeze valve having the additional behavior of being a flow regulation device. Each 3D configuration was made possible through our specialized DLP-SLA 3D-printing processes. We integrated each valve type into micropumps and were able to determine the maximum achievable flow rates for each design. We also determined the geometric and operational constraints of using ribbon valve pumps in parallel for diffusion mixing and demonstrated their effectiveness.

The results presented in this paper show that both flow-driven and pressure-driven nanoliter-scale passive nonlinear microfluidic components can be fabricated using 3D printing. We have also shown that these components can be integrated into higher-level systems such as pumps and mixers which are simpler to drive than counterparts consisting entirely of active components. Such advantages should help facilitate the adoption of 3D-printing technology for microfluidics.

## Data Availability

The data presented in this study are available on request from the corresponding author.

## Supplementary Materials

The following supporting information can be downloaded at: https://www.mdpi.com/article/10.3390/mi14071286/s1. Figure S1: Photographs of 3D printed chips for one-way valve component testing. (a) Chip with 7 squeeze one-way valves configured to be individually tested. Each valve inlet and outlet is routed to separate PTFE tubes glued to the chip to serve as chip-to-world fluid connections. (b) Close-up photo of chip in (a). (c) Three simultaneously 3D printed chips, each with a single one-way valve-based pump. (d) Multiplexed one-way valve test components that are 3D printed in a single chip. Video S1: Flow in the channels and one-way valve is induced manually with a syringe filled with red-colored water connected with PTFE tubing to a one-way valve test chip. The syringe plunger is alternately pushed and pulled to infuse and extract fluid from the device. Fluid flow in the forward direction results in the membrane being pushed up in the video such that fluid flows freely. Fluid flow in the reverse direction causes the membrane to deflect downward and close off the lower channel, thereby preventing fluid flow. Video S2: Operational parameters for the membrane pump video are 60 ms phase time, Δt; DC applied pressure of 20 psi to actuate DC membrane during one phase time; and −23 inHg DC applied vacuum during other phase time. The pump is located to the left side of the serpentine channel out of the field of view of the microscope such that the entire serpentine channel fields in the microscope field of view in order to clearly see the full fluid flow.
