# Supplementary material for: 3D-Printed Microfluidic One-Way Valves and Pumps
Source: Micromachines (Basel). 2023 Jun 23;14(7):1286. doi: 10.3390/mi14071286 (PMC10384158; doi:10.3390/mi14071286)
Supplement: Supplementary file 1 [file micromachines-14-01286-s001.zip › Supplementary_Information.pdf]

3D Printed Microfluidic One-Way Valves and Pumps

Hunter Hinnen[1], Matthew Viglione[1], Troy R. Munro[2], Adam T. Woolley[3], and Gregory P. Nordin[1]

[1]Electrical and Computer Engineering, [2]Mechanical Engineering, [3]Chemistry & Biochemistry

Supplementary Information

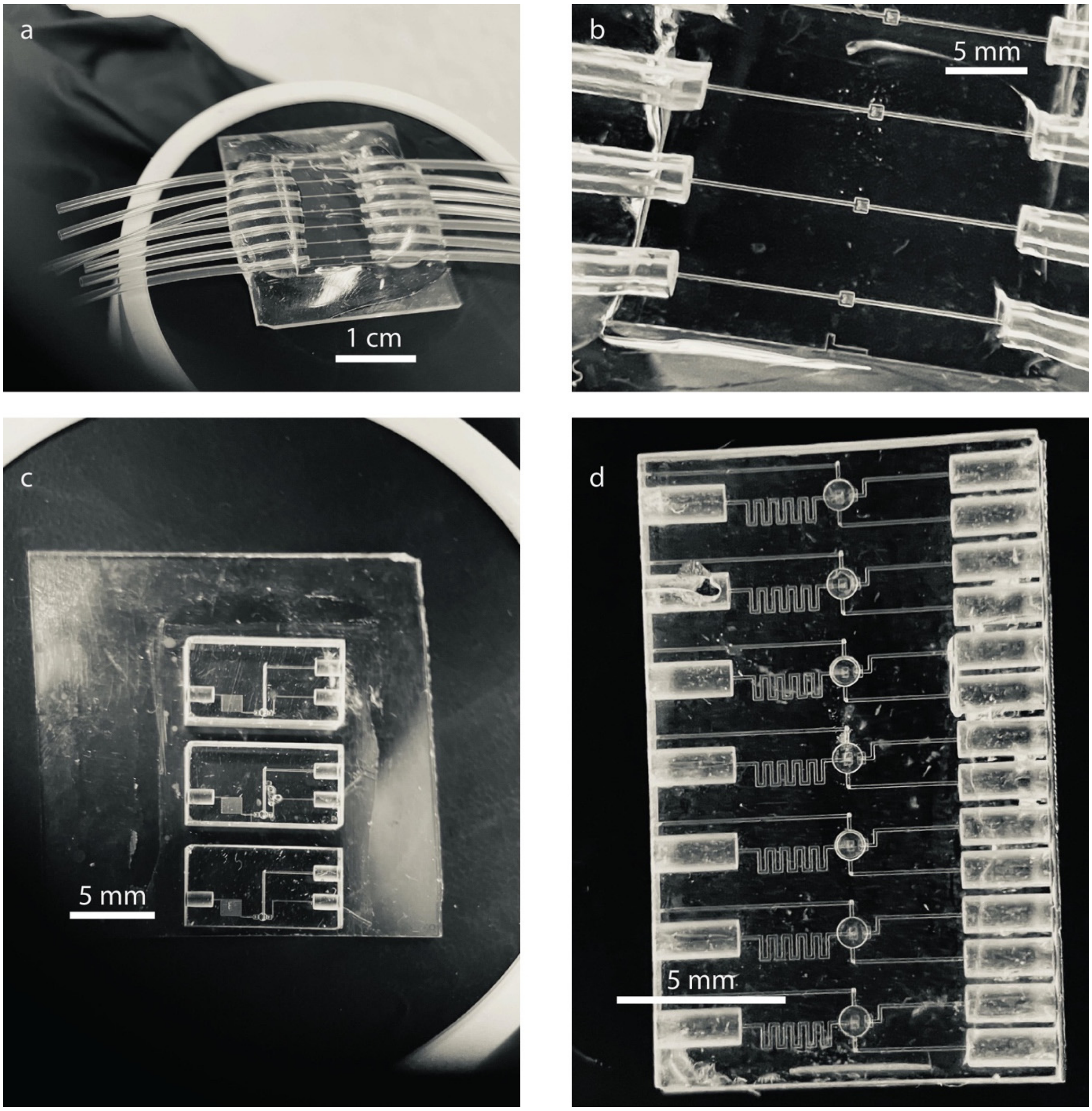

Figure S1. Photographs of 3D printed chips for one-way valve component testing. (a) Chip with 7 squeeze one-way valves configured to be individually tested. Each valve inlet and outlet is routed to separate PTFE tubes glued to the chip to serve as chip-to-world fluid connections. (b) Close-up photo of chip in (a). (c) Three simultaneously 3D printed chips, each with a single one-way valve-based pump. (d) Multiplexed one-way valve test components that are 3D printed in a single chip.

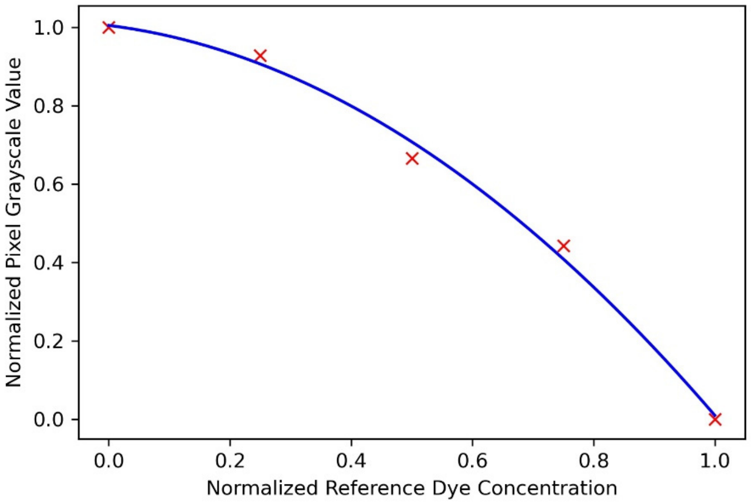

Figure S2. Normalized calibration curve for pixel grayscale value as a function of black dye concentration.

Video S1—Membrane One-Way Valve

Flow in the channels and one-way valve is induced manually with a syringe filled with red-colored water connected with PTFE tubing to a one-way valve test chip. The syringe plunger is alternately pushed and pulled to infuse and extract fluid from the device. Fluid flow in the forward direction results in the membrane being pushed up in the video such that fluid flows freely. Fluid flow in the reverse direction causes the membrane to deflect downward and close off the lower channel, thereby preventing fluid flow.

Video S2—Membrane Pump

Operational parameters for the membrane pump video are 60 ms phase time, Δt; DC applied pressure of 20 psi to actuate DC membrane during one phase time; and -23 inHg DC applied vacuum during other phase time. The pump is located to the left side of the serpentine channel out of the field of view of the microscope such that the entire serpentine channel fields in the microscope field of view in order to clearly see the full fluid flow.

Measured Concentration Calibration Curve
